# Editorial: The impact of COVID-19 on food security around the world

**DOI:** 10.3389/fpubh.2023.1271241

**Published:** 2023-08-23

**Authors:** Carolina Abreu de Carvalho

**Affiliations:** Post-Graduate Program in Public Health, Federal University of Maranhão, São Luís, MA, Brazil

**Keywords:** food insecurity, COVID-19 pandemic, population health, public policy, post-pandemic COVID-19

The COVID-19 pandemic, in addition to the measures adopted to prevent its spread (such as social isolation, closure of schools and businesses, travel restrictions, and blockages), were responsible for the increase in the prevalence and severity of food insecurity throughout the world. The pandemic has had direct and indirect impacts on global health and economy, the pattern of consumption, and the way food is produced and distributed.

The Research Topic published by Frontiers in Public Health “*The impact of COVID-19 on food security around the world*” aimed to understand the repercussions of COVID-19 on food insecurity, gathering articles that can support the implementation of new public health policies to combat food insecurity that worsened after the COVID-19 pandemic. This Research Topic of six articles brought together studies from different countries in the world (China, Peru, United States, Brazil), with different socioeconomic contexts, but which despite these differences have the similarity of having been widely affected by the COVID-19 pandemic, especially regarding the state of food security of its population.

One of the most drastic consequences of COVID-19 pandemic was inflation. Around the world food prices have soared and directly impacted food access, especially by the most vulnerable groups. Leung and Wolfson showed this by studying the short-term effects of increasing the benefit of the Supplemental Nutrition Assistance Program (SNAP), the largest nutritional assistance program in the United States. The rising food prices and growing inflation potentially attenuated the benefits of the policy change on participants' outcomes. Similar results were observed by Mota et al. in Brazilian university students benefiting from a student assistance program. About 45% reported that the aid offered by the university was the family's only source of income during the pandemic and 65% used it to buy food. However, even with this aid, more than half described a worsening in food quality, related to food prices. Therefore, it is essential that governments develop actions to control inflation and focus on food security policies that aim to protect, especially the most vulnerable population, against rising food prices, which is one of the main causes of reduced access to food.

Another aspect highlighted in this Research Topic is the importance of considering that the impacts of the COVID-19 pandemic on the food security of populations, unfortunately, are not isolated. Countries and their populations have been living with the combination of risk factors for food security that add to the effects of the pandemic and generate an even more devastating scenario for the state of food security of the population. This is what we see in the study by Valladares-Garrido et al., who found a high prevalence of food insecurity during the co-occurrence of the COVID-19 pandemic and the earthquake that occurred in July 2021 in the region of Piura, northern Peru. It is interesting to note that the prevalence of food insecurity was even higher in those families that had experienced the effects of the El Nino phenomenon in the region in 2017. This is a true syndemic of poverty, natural disasters, the COVID-19 pandemic and food insecurity, factors that interact, and have an exacerbated effect on the food security of the population.

It is known that the harmful effects of food insecurity are potentiated when they affect children. Azevedo et al., in a systematic review and meta-analysis, investigated the main factors associated with food insecurity among pregnant women or mothers of children under 2 years of age during the pandemic. The authors observed that food insecurity was associated with factors such as domestic violence, mental health problems, low socioeconomic status, unemployment, and supply chain disruptions. It is a major public health concern to try to mitigate the profound impacts that the COVID-19 pandemic, combined with food insecurity, can have on an entire generation of children, especially those in the first one thousand days of life.

All the experiences lived in recent years with the COVID-19 pandemic and its impacts on food insecurity must be analyzed by policymakers in order to prevent or mitigate greater damages in similar situations in the future. In this regard, Ding et al. propose a food safety regulation model based on the joint efforts of the public authorities and all sectors of society to promote the development of food safety regulations, creating a solid foundation for the prevention and control of major public crises.

Schanbacher and Cavendish report important lessons from community gardens in Florida for promoting food security and overall community wellbeing. The establishment of clear emergency protocols, efficient use of resources, maintenance of educational programs, strengthening communications, and cooperation with other gardens and actors in local food supply chains are some of the suggestions highlighted by the authors of this article as ways to help ensure food security in the context of community gardens.

Certainly, the impacts of the COVID-19 pandemic on food insecurity are still felt around the world, as they have been profound, widespread, and complex. However, governments and societies urgently need to organize and expedite the implementation of multisectoral policies that can directly address these impacts, especially for the most vulnerable groups.

## Author contributions

CC: Conceptualization, Writing—original draft, Writing—review and editing.

